# THE ROLE OF MULTIDOMAIN IDENTITY AND SOCIAL BACKGROUND FACTORS FOR AGING OUTCOMES

**DOI:** 10.1093/geroni/igac059.690

**Published:** 2022-12-20

**Authors:** Johanna Drewelies, Sandra Duezel, Ilja Demuth, Elisabeth Steinhagen-Thiessen, Ulman Lindenberger, Jan Goebel, Gertraud Stadler, Denis Gerstorf

**Affiliations:** Humboldt University Berlin, Berlin, Berlin, Germany; Max-Planck-Insitute for human developement, Berlin, Berlin, Germany; Charité Universitätsmedizin Berlin, Berlin, Berlin, Germany; Charité - Universitätsmedizin Berlin, Berlin, Berlin, Germany; Max Planck Institute for Human Development, Berlin, Berlin, Germany; German Institute for Economic Research, Berlin, Berlin, Germany; Charité – Universitätsmedizin Berlin, Berlin, Berlin, Germany; Humboldt Universität zu Berlin, Berlin, Berlin, Germany

## Abstract

Domains of social privilege are predictive of a number of key aging outcomes, such as physical and cognitive functioning. However, less is known about the dynamic interplay of - related but distinct- domains of privilege and how they are associated with cognitive and physical functioning. In the following project, we aim to close this gap by examining the role of multidomain identity and social background factors for initial levels and subsequent changes in psychological, cognitive and physical functioning. Specifically, we identified predefined social privileges within the Berlin Aging Study II (BASE-II) sample (e.g., gender identity, gender conformity, sexuality, education, socioeconomic status, religion) and related these categories, interactively, to markers of physical health (multimorbidity), perceptual speed (digit symbol), and psychosocial functioning (loneliness). Results highlight the dynamic interplay (i.e., intersectionalism) of multidomain identity and social background factors with aging related outcomes across a number of key domains.

